# Effect of Copper Sulfate and Sulfuric Acid on Blind Hole Filling of HDI Circuit Boards by Electroplating

**DOI:** 10.3390/ma14010085

**Published:** 2020-12-27

**Authors:** Pingjun Tao, Yugan Chen, Weitong Cai, Zhaoguang Meng

**Affiliations:** 1 School of Materials and Energy, Guangdong University of Technology, Guangzhou 510006, China; Yg_Chen001@163.com (Y.C.); mewtcai@gdut.edu.cn (W.C.); 2 Dongguan Wuzhu Electronic Technology Co., Ltd., Dongguan 523390, China; skill@topcb.com.cn

**Keywords:** HDI printed circuit board, electroplating, blind hole filling, sag degree, copper plating layer

## Abstract

Here, in a certain high density interconnect (HDI) printed circuit board, the effect of copper sulfate and sulfuric acid on the filling effect of a blind hole with a certain diameter and depth was investigated by making a blind hole using a CO_2_ laser drilling machine, filling the blind hole via electroplating by simulating the electroplating line in a Halin cell, and observing the cross-section of a micro blind hole after polishing using metallographic microscope, as well as the effect of hole filling, are evaluated. The results show that, under the conditions of a certain plating solution formula and electroplating parameters (current density and electroplating time), the sag degree decreases with the increase in the copper sulfate concentration. When the concentration of copper sulfate increases from 210 g/L to 225 g/L, the filling effect is good and the sag degree is about 0. However, with the increase in sulfuric acid concentration, the sag increases gradually. When the sulfuric acid concentration is 25–35 g/L, both the sag and copper coating thickness are in a small range. Under appropriate electroplating conditions, a better blind hole filling effect can be obtained. The volume of blind hole has a certain effect on the diffusion and exchange of copper sulfate and sulfuric acid, as well as on the concentration distribution of additives.

## 1. Introduction

High density interconnect (HDI) printed circuit boards were proposed in the United States and Japan in the early 1990s. This kind of printed circuit board is also known as a “build-up multilayer” board. HDI is a fine circuit, small aperture, high density, and ultra-thin printed circuit board [1,2,3]. Its main features are as follows: the aperture is not more than 0.15 mm, the hole ring diameter is less than 0.25 mm, the density of welding point is greater than 0.2 points/mm^2^ or 130 points/in^2^, the wiring density is greater than 4.6 mm/mm^2^ or 117 in/in^2^, and the line/space is not more than 0.076 mm/0.076 mm or 3 mil/3 mil (1 mil = 0.254 mm). With the development of electronic equipment moving in the direction of being light, thin, intelligent, and multi-functional, HDI is required to have high-density interconnection and high reliability. Blind hole electroplating, which has been widely studied, is one of the important technologies in order to realize the high-density interconnection and high reliability of printed circuit boards [4]. The basic principle of blind hole filling in electroplating is to fill the blind hole with super filling electrodeposition mode, i.e., bottom-up deposition mode. In the bottom-up deposition mode, the deposition rate of copper on the bottom of the blind hole is greater than that on the surface of the plate, so as to achieve the filling effect of having no cavity, a low sag, and a low surface copper plating thickness [5,6]. However, although electroplating blind hole filling technology has been applied in the field of HDI printed circuit board manufacturing, how to implement high-quality blind hole electroplating filling of an HDI printed circuit board is still a research hotspot [7,8,9,10].

A large number of studies have shown that there are many factors affecting the effect of blind hole filling, such as the formula of the electroplating solution (composition and concentration), electroplating process parameters (electroplating method, stirring mode, current density, and electroplating time), and the geometric size of blind hole (diameter and diameter depth ratio) [11,12,13]. However, there are not many reports about how to obtain a good blind hole filling effect for an HDI substrate by changing the content and proportion of copper sulfate and sulfuric acid in the plating solution formula, and the available references are very limited. Therefore, in order to improve the quality of blind hole filling and to improve the production process of blind hole filling in HDI printed circuit board electroplating, this paper takes a blind hole with a specific hole depth and aperture as the research object, and studies the influence of copper sulfate and sulfuric acid in the plating solution formula on the blind hole filling effect, so as to lay the foundation for optimizing the blind hole filling electroplating process and plating solution formula, as well as realizing high-quality blind hole filling electroplating.

## 2. Materials and Methods

### 2.1. Materials and Instruments

The following were used: copper sulfate CuSO_4_·5H_2_O, analytical pure; sulfuric acid (96–98%), analytical pure; hydrochloric acid (38–40%), analytical pure; electroplating solution additives (including wetting agent, leveling agent, and brightener) for blind hole filling in electroplating, provided by Dongguan Wuzhu Electronic Technology Co., Ltd., Dongguan, China; FR4 (Abbreviation of flame retardant type 4) copper clad laminate, provided by Dongguan Wuzhu Electronic Technology Co., Ltd.; and distilled water, self-made. The following equipment were used: HD600E2A CO_2_ dual beam dual table PCB laser drilling machine, Han’s Laser Technology Industry Group Co., Ltd., Shenzhen, China; Horizontal Orizontal Electroless Copper Line, Guangzhou Julong PCB Equipment Co., Ltd., Guangzhou, China; vertical continuous electroplating production line, Yongtian Machinery Equipment Manufacture (Shenzhen) Co., Ltd., Shenzhen, China; Haring cell, Suzhou Jiangdong Precision Instrument Co., Ltd., Suzhou, China; DC (Abbreviation of direct current) rectifier, Xiamen Songye Electric Technology Co., Ltd., Xiamen, China; JMP-2B metallographic slicing grinder, Shenzhen Jujie Instrument Equipment Co., Ltd., Shenzhen, China; and metallographic microscope, Shanghai Wumo Optical Instrument Co., Ltd., Shanghai, China.

### 2.2. Preparation of Hole-Filling Plate

In this paper, 12 μm copper foil, 106 pp/12 μm copper foil, and 1080 pp laminate were selected, and blind holes with diameters of 75 μm, 100 μm, and 125 μm were made using a CO_2_ laser drilling machine on double-sided plates with a medium layer thickness of 60 μm and 80 μm, respectively, and the distance between holes was 500 μm. The thickness of the copper coating was about 5 μm using chemical deposition and flash plating. The plates were cut into 100 mm × 50 mm test plates, which were degreased, washed, micro etched, washed, and pickled.

### 2.3. Electroplating

In a 500-mL Halin cell, the experiment plates were electroplated with a simulated electroplating line using DC plating. The anode was an insoluble anode, the stirring mode was air stirring, and the distance between the anode and cathode was 0.5 cm. The bath temperature was 25 degrees centigrade. The concentration control range of each component in the electroplating solution was as follows: 200~260 g/L CuSO_4_·5H_2_O, 20~60 g/L H_2_SO_4_, 2~22 mL/L wetting agent, 0~2 mL/L sulfur containing brightener, 5~25 mL/L nitrogen containing leveling agent, and 30~70 mg/L Cl^−^.

### 2.4. Characterization of Blind Hole-Filling Effect

**Figure 1:**
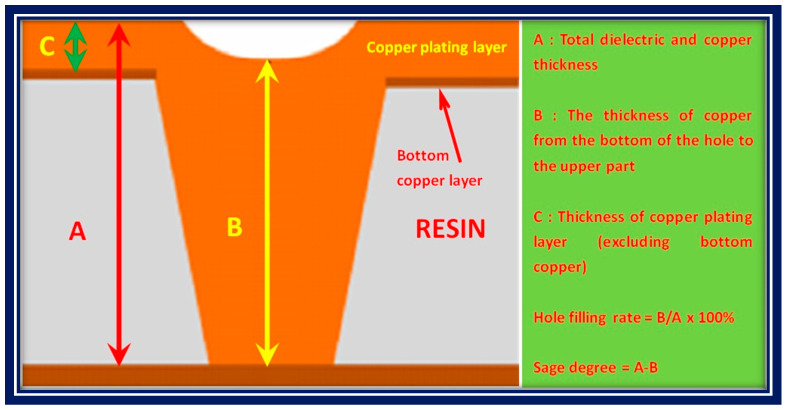
Characterization of the blind hole filling capacity.

The cross section of the micro blind hole after polishing was observed using a metallographic microscope. The filling effect of the hole filling electroplating is usually characterized by the filling rate, copper thickness, and sag degree. The hole filling rate = B/A × 100% and the sag degree = A − B, where A is the surface thickness of the copper layer from the bottom of the blind hole to the surface of the plate, B is the thickness of the most concave copper layer from the hole bottom of the blind hole to the surface of the hole, and C is the thickness from the copper foil to the surface of the copper layer on the plate surface, as shown in Figure 1. According to whether the blind hole is fully filled; whether there are holes, cavities, and other defects in the hole; and whether the filling rate meets certain industry requirements, the filling effect can be judged. The sag degree should be controlled within a dozen microns. The thickness of the copper plating should be as small as possible on the premise of ensuring that the sag meets the production requirements.

## 3. Results and Discussion

The process of hole filling electroplating must maximize the filling capacity and minimize the thickness of the surface copper, showing comprehensive characteristics of a low sag, uniform copper surface distribution, and no cavity. The filling capacity of electroplating can be characterized by the sag degree, copper plating thickness, and filling rate. Figure 1 shows the characterization of the blind hole filling capacity. In order to meet the high density interconnection requirements of HDI circuit boards, the effect of blind hole filling by electroplating is generally evaluated from the following aspects. The first requirement is that the sag should not exceed 15 μm. The second requirement is that the deposition thickness of the copper should not exceed 25 μm. The third requirement is that the filling rate should be greater than 78%. The fourth requirement is that the copper coating should be even and smooth. However, the electroplating process conditions, the composition and formula of the electroplating solution, and the level of the electroplating hole filling process will have more or less influence on the filling effect.

**Figure 2:**
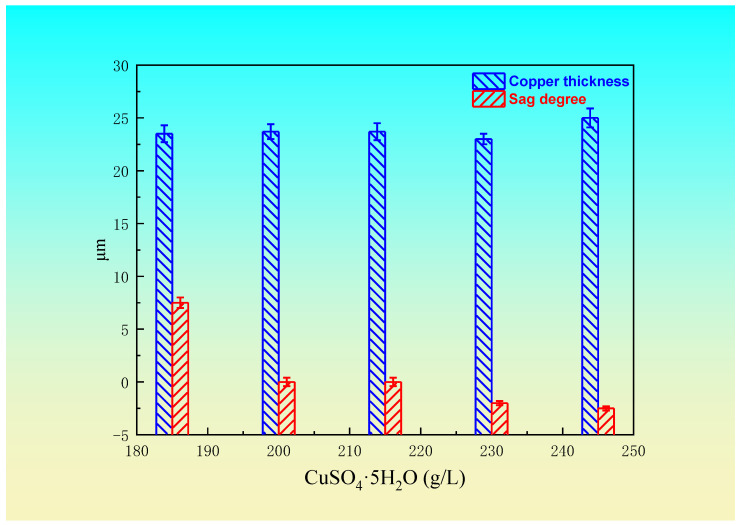
Effect of the copper sulfate concentration on the sag degree and copper thickness.

Copper sulfate is the main source of copper ions in an electroplating solution. A high copper and low acid system is usually used in electroplating hole filling. When the concentration of copper sulfate is too low, the deposition rate of copper is slow. When the concentration of copper sulfate is too high, the crystal particles increase and affect the dispersion ability of the electroplating solution. Figure 2 shows the relationship between the copper sulfate concentration and the blind hole filling effect (35 g/L H_2_SO_4_, 45 mg/L Cl^−^, 15 mL/L leveling agent, 1 mL/L brightener, 10 mL/L wetting agent, 1.5 A/dm^2^ current density, and 55 min electroplating time). It can be seen from Figure 2 that the sag decreases with the increase in the copper sulfate concentration. When the CuSO_4_·5H_2_O concentration increases from 210~225 g/L, the filling effect is good and the sag is about 0. When the concentration of CuSO_4_·5H_2_O increases to 230 g/L and above, the blind hole filling is prominent and overfilled, and CuSO_4_·5H_2_O is easy to crystallize and precipitate. On the other hand, the thickness of the copper plating on the surface is almost independent of the concentration of the copper sulfate, and the thickness of copper is between 19 and 24 μm, which indicates that the concentration of copper sulfate hardly affects the thickness of the copper in the experimental range. The results from the above experimental data show that the blind hole filling effect was good when the concentration of CuSO_4_·5H_2_O varied from 210 to 225 g/L.

**Figure 3:**
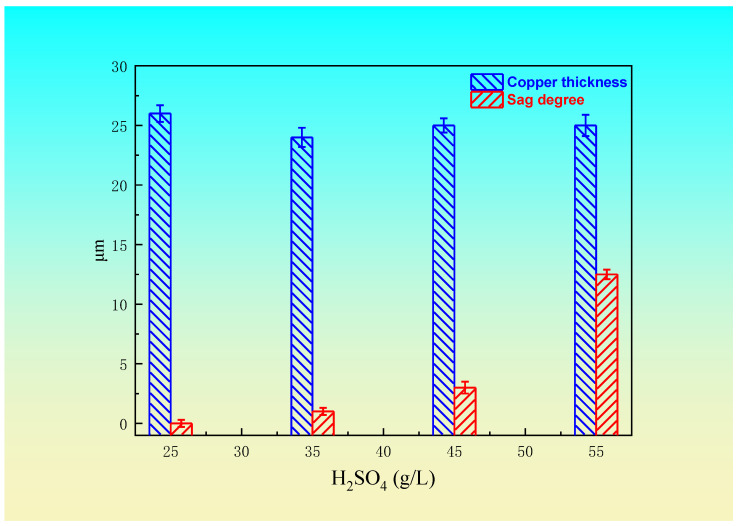
Effect of the sulfuric acid concentration on the sag and thickness.

The concentration of sulfuric acid is one of the most important factors affecting the effect of blind hole filling. The increase in sulfuric acid concentration can improve the conductivity and dispersion of the electroplating solution. However, if the sulfuric acid concentration is too high, the leveling agent will be protonated rapidly, which will affect the electroplating effect, and the ductility of the copper coating will be reduced. When the sulfuric acid concentration is too low (<25 g/L), the blind hole filling mode is of an equiangular deposition, which is not conducive to blind hole filling. The relationship between the sulfuric acid concentration and blind hole filling effect is shown in Figure 3 (210 g/L CuSO_4_·5H_2_O, 45 mg/L Cl^−^, 15 mL/L leveling agent, 1 mL/L brightener, 10 mL/L wetting agent, 1.5 A/dm^2^ current density, and 55 min electroplating ). It can be seen from Figure 3 that when the sulfuric acid concentration is greater than 25 g/L, with the increase of sulfuric acid concentration, the thickness of copper plating layer changes slightly, but the sag gradually increases. When the concentration of sulfuric acid is 25~35 g/L, the sag and the thickness of the copper coating are small. Therefore, the results of the above experimental data show that the blind hole filling effect was good when the optimum concentration of sulfuric acid was 25~35 g/L.

**Figure 4:**
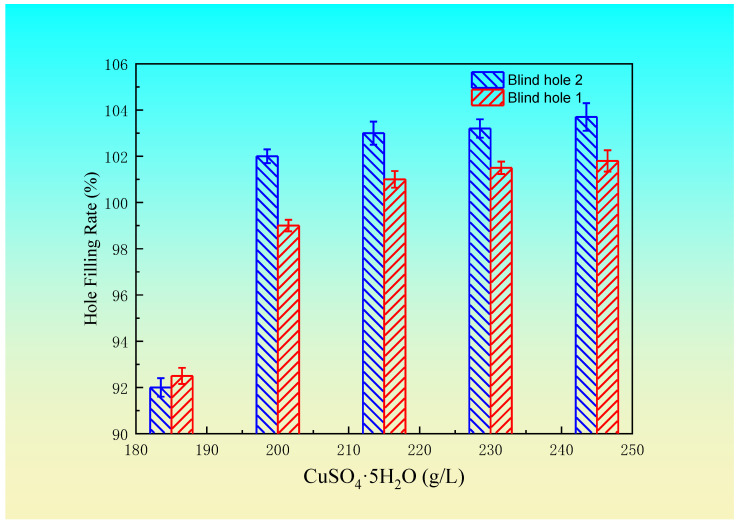
Change in the hole filling rate with the copper sulfate concentration.

**Figure 5:**
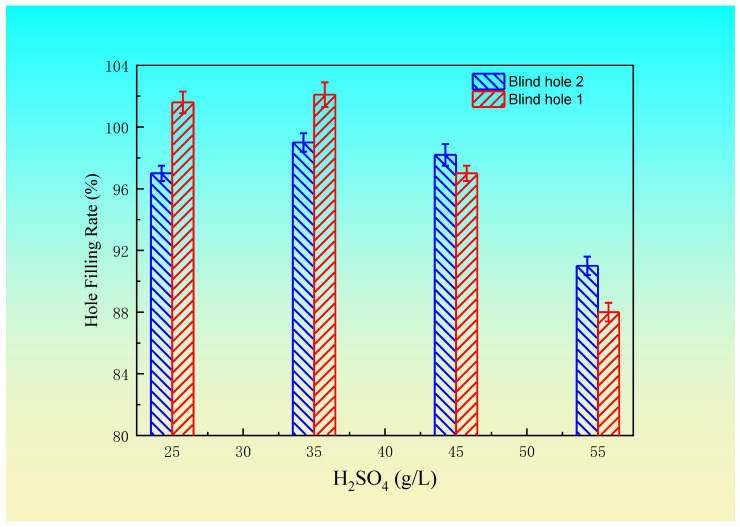
Change in the hole filling rate with sulfuric acid concentration.

Based on the above test results, the suitable electroplating process and bath formula for a blind hole with a diameter of 100 μm and depth of 50 μm are as follows: 210~225 g/L CuSO_4_·5H_2_O concentration, 25~35 g/L H_2_SO_4_ concentration, 45~60 mg/L Cl^−^ concentration, 0.8~1.2 mL/L brightener concentration, 10~16 mL/L leveling agent, 50~80 min electroplating time, 10~15 mL/L wetting agent concentration, and 1.4~1.7 A/dm^2^ current density. According to the selected plating solution formula, blind hole 1 (depth diameter ratio of 1:2), with a diameter of 100 μm and a depth of 50 μm, and blind hole 2 (depth diameter ratio of 1.17:1), with a hole diameter of 60 μm and depth of 70 μm, were each plated under different levels of various factors. The optimum concentration of sulfuric acid was obtained by fixing the H_2_SO_4_ at a concentration of 25 g/L, Cl^−^ concentration of 45 mg/L, wetting agent concentration of 10 mL/L, brightener concentration of 1 mL/L, leveling agent of 15 mL/L, current density of 1.5 A/dm^2^, and electroplating time of 55 min, and changing the concentration of the copper sulfate. Then, by selecting the best concentration of copper sulfate, while the other factors remain unchanged, the best concentration of sulfuric acid could be obtained through experiments. The hole filling rate changes of the two types of holes are shown in Figure 4 and Figure 5, respectively.

**Figure 6:**
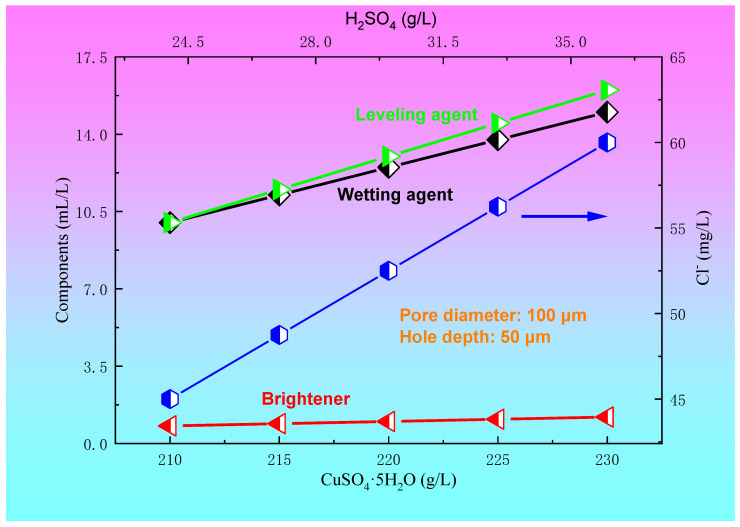
The relationships between the concentration of CuSO_4_·5H_2_O and H_2_SO_4_ and the plating solution formula for the blind hole with a diameter of 100 μm and a depth of 50 μm.

**Figure 7:**
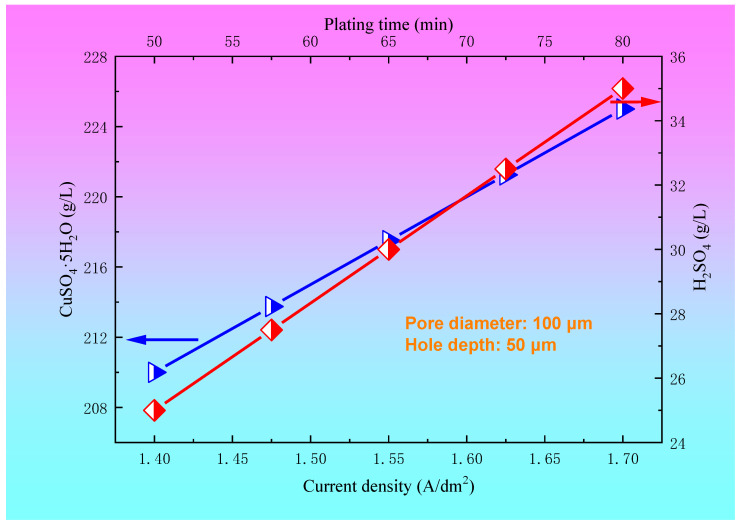
The relationships between the current density and electroplating time and the concentration of CuSO_4_·5H_2_O and H_2_SO_4_ for the blind hole with a diameter of 100 μm and a depth of 50 μm.

The relationships between the concentration of CuSO_4_·5H_2_O and H_2_SO_4_ and the plating solution formula for the blind hole with a diameter of 100 μm and a depth of 50 μm are shown in Figure 6. For the blind hole with a diameter of 100 μm and a hole depth of 50 μm, within a certain range of plating solution formula, such as a 45~60 mg/L Cl^−^ concentration, 0.8~1.2 mL/L brightener concentration, 10~16 mL/L leveling agent, and wetting agent concentration of 10~15 mL/L, copper sulfate and sulfuric acid had a preferred region to achieve better filling effect. In the present paper, the optimum ranges of the concentrations of copper sulfate and sulfuric acid were 210~225 g/L and 25~35 g/L, respectively. The relationships between the current density and electroplating time and the concentration of CuSO_4_·5H_2_O and H_2_SO_4_ for a blind hole with a diameter of 100 μm and a depth of 50 μm are shown in Figure 7. It can be seen from Figure 7 that when copper sulfate and sulfuric acid are located in the preferred regions, the process conditions for achieving a good filling effect are a current density of 1.4~1.7 A/dm^2^ and electroplating time of 50~80 min.

**Figure 8:**
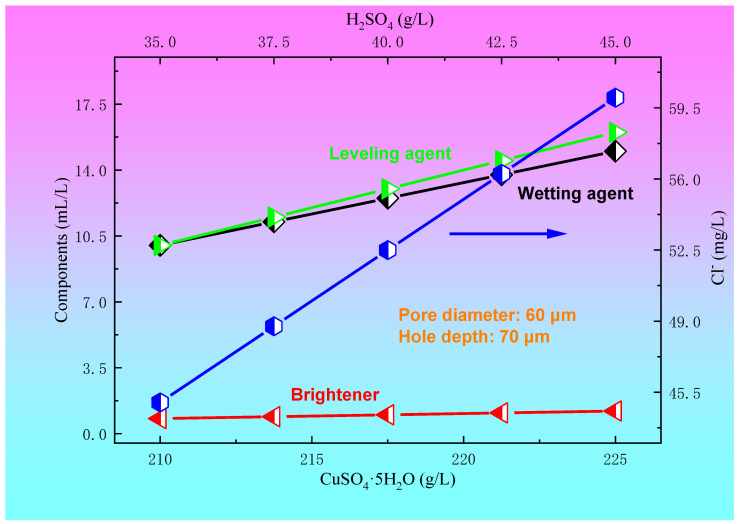
The relationships between the concentration of CuSO_4_·5H_2_O and H_2_SO_4_ and the plating solution formula for the blind hole with a diameter of 60 μm and a depth of 70 μm.

**Figure 9:**
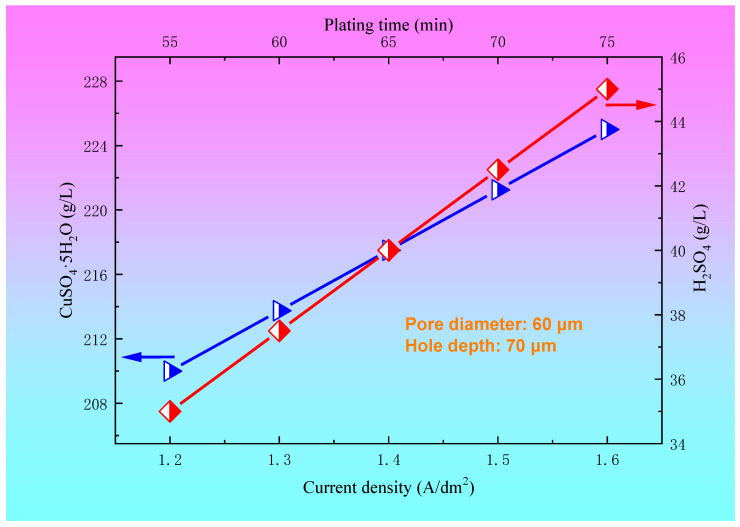
The relationships between the current density and electroplating time and the concentration of CuSO_4_·5H_2_O and H_2_SO_4_ for the blind hole with a diameter of 60 μm and a depth of 70 μm.

The relationships between the concentration of CuSO_4_·5H_2_O and H_2_SO_4_ and the plating solution formula for a blind hole with a diameter of 60 μm and a depth of 70 μm are shown in Figure 8. For a blind hole with a diameter of 60 μm and a hole depth of 70 μm, within a certain range of the plating solution formula, such as a Cl^−^ concentration of 45~60 mg/L, brightener concentration of 0.8~1.2 mL/L, leveling agent of 10~16 mL/L, and wetting agent concentration of 10~15 mL/L, copper sulfate and sulfuric acid also have a preferred region in order to achieve a better filling effect. In the present paper, the optimum ranges of the concentration of CuSO_4_·5H_2_O and H_2_SO_4_ were 210~225 g/L and 35~45 g/L, respectively. The relationships between the current density and electroplating time and the concentration of CuSO_4_·5H_2_O and H_2_SO_4_ for a blind hole with a diameter of 60 μm and a depth of 70 μm are shown in Figure 9. It can be seen from Figure 9 that when copper sulfate and sulfuric acid are located in the preferred regions, the process conditions for achieving a good filling effect are a current density of 1.2~1.6 A/dm^2^ and electroplating time of 55~75 min.

**Figure 10:**
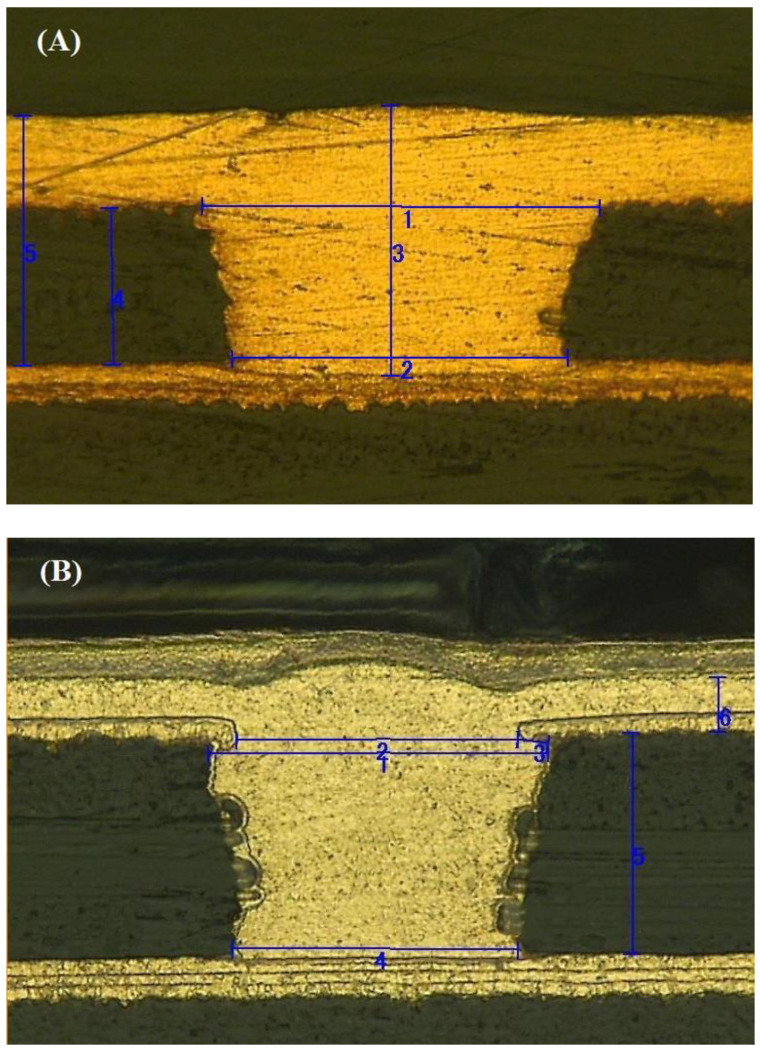
The representative section of metallographic micrographs of the blind holes. (**A**) A blind hole with a diameter of 100 μm and a hole depth of 50 μm, and (**B**) a blind hole with a diameter of 60 μm and a hole depth of 70 μm.

The section morphology of the micro blind hole after polishing was observed using a metallographic microscope. For a blind hole with a diameter of 100 μm and a hole depth of 50 μm, when the current density was 1.4~1.7 A/dm^2^ and the electroplating time was 50~80 min, the representative metallographic micrographs of the blind hole are shown in Figure 10A. For the blind hole with a diameter of 60 μm and a hole depth of 70 μm, when the current density was 1.2~1.6 A/dm^2^ and the electroplating time was 55~75 min, the representative metallographic micrographs of the blind hole are shown in Figure 10B. As can be seen from Figure 10, it was found that there were no holes, pores, contaminants, and delamination fractures in the blind holes, showing good filling effects.

The overall trend of the filling rate for the two blind holes with CuSO_4_·5H_2_O and H_2_SO_4_ concentrations is similar, but there are differences due to different thickness the diameter ratio. The hole filling rate is different under different concentrations of CuSO_4_·5H_2_O and H_2_SO_4_. Different sizes of blind holes require different concentrations of CuSO_4_·5H_2_O and H_2_SO_4_, and the electroplating conditions are also different. The volume of the blind hole has a certain effect on the diffusion exchange of CuSO_4_·5H_2_O and H_2_SO_4_ and the concentration distribution of the additives. Different sizes of blind holes require different electroplating conditions. When the size of the blind holes changes, the concentration of CuSO_4_·5H_2_O and H_2_SO_4_ in the electroplating solution should also change accordingly. Therefore, when filling a blind hole, the proper electroplating conditions should be selected according to the hole geometry size, so as to obtain an excellent filling hole.

## 4. Conclusions

Under the conditions of a certain plating solution formula and electroplating parameters (current density and electroplating time), the sag degree decreases with the increase in the copper sulfate concentration. When the concentration of copper sulfate increases from 210 g/L to 225 g/L, the filling effect is good, and the sag degree is about 0. However, with the increase of sulfuric acid concentration, the sag increases gradually. When the sulfuric acid concentration is 25–35 g/L, both the sag and copper coating thickness are in a small range. For a blind hole with pore diameter of 100 μm and hole depth of 50 μm, under the process conditions of a 1.6~1.8 A/dm^2^ current density and 55~75 min electroplating time, when the concentrations of CuSO_4_·5H_2_O and H_2_SO_4_ are 210~225 g/L and 25~35 g/L, respectively, a better filling effect can be obtained. For a blind hole with a pore diameter of 60 μm and hole depth of 70 μm, under the process conditions of a 1.4~1.7 A/dm^2^ current density and 60~75 min electroplating time, when the concentrations of CuSO_4_·5H_2_O and H_2_SO_4_ are 210~225 g/L and 35~45 g/L, respectively, a better filling effect can be obtained. The volume of the blind hole has a certain effect on the diffusion and exchange of CuSO_4_·5H_2_O and H_2_SO_4_, as well as on the concentration distribution of additives.

## Data Availability

The authors confirm that the data supporting the findings of this study are available within the article and the referenced article.
